# Long-term health-related quality of life of critically ill patients with haematological malignancies: a prospective observational multicenter study

**DOI:** 10.1186/s13613-018-0478-3

**Published:** 2019-01-05

**Authors:** Franck Ehooman, Lucie Biard, Virginie Lemiale, Damien Contou, Nicolas de Prost, Djamel Mokart, Frédéric Pène, Achille Kouatchet, Julien Mayaux, Alexandre Demoule, François Vincent, Martine Nyunga, Fabrice Bruneel, Antoine Rabbat, Christine Lebert, Pierre Perez, Anne-Pascale Meert, Dominique Benoit, Rebecca Hamidfar, Michael Darmon, Elie Azoulay, Lara Zafrani

**Affiliations:** 1 Medical ICU, Saint-Louis Teaching Hospital, 1 Avenue Claude Vellefaux, 75010 Paris, France; 2 Biostatistics Department, Saint-Louis Teaching Hospital, Paris, France; 3 Medical ICU, Henri Mondor Teaching Hospital, Paris, France; 4 ICU, Paoli Calmette Institute, Marseille, France; 5 Medical ICU, Cochin Teaching Hospital, Paris, France; 6 Medical ICU, Angers Teaching Hospital, Angers, France; 7 Medical ICU, Pitié-Salpétrière Teaching Hospital, Paris, France; 8 ICU, Avicenne Teaching Hospital, Bobigny, France; 9 ICU, Roubaix Hospital, Roubaix, France; 10 ICU, Mignot Hospital, Versailles, France; 11 Respiratory Unit, Cochin Teaching Hospital, Paris, France; 12 ICU, Vendée hospital, La Roche sur Yon, France; 13 ICU, Brabois Teaching Hospital, Nancy, France; 14 Service soins intensifs et urgences oncologiques, Institut Jules Bordet, Brussels, Belgium; 15 ICU, Ghent Teaching Hospital, Ghent, Belgium; 16 ICU, Albert Michallon University Hospital, Grenoble, France; 17 ICU, Saint-Etienne University Hospital, Saint-Etienne, France

**Keywords:** Intensive care unit, Haematological malignancy, Long-term health-related quality of life, Anxiety, Depression, Post-traumatic syndrome disorder

## Abstract

**Background:**

Although outcomes of critically ill patients with haematological malignancies (HMs) have been fully investigated in terms of organ failure and mortality, data are scarce on health-related quality of life (HRQOL) in this population. We aim to assess post-intensive care unit (ICU) burden and HRQOL of critically ill patients with HMs and to identify risk factors for quality-of-life (QOL) impairment.

**Results:**

In total, 1011 patients with HMs who required ICU admission in 17 ICUs in France and Belgium were included in the study; 278 and 117 patients were evaluated for QOL at 3 months and 1 year, respectively, after ICU discharge. HRQOL was determined by applying the interview form of the Short Form 36 (SF-36) questionnaire. Psychological distress symptoms were evaluated using the Hospital Anxiety Depression Score (HADS) and the Impact of Event Scale (IES). In-hospital mortality rates at 3 months and 1 year were, respectively, 39.1, 50.7 and 57.2%, respectively. At 3 months, median [IQR] physical and mental component summary scores (PCS and MCS) (SF-36) were 37 [28–46] and 51 [45–58], respectively. PCS was lower in ICU patients with HMs when compared to general ICU septic patients (52 [5–13], *p* = 0.00001). The median combined HAD score was 8 [5–13], and the median IES score was 8 [3–16]. However, recovery during the first year after ICU discharge was not consistent in all dimensions of HRQOL. Three months after ICU discharge, the maximum daily Sequential Organ Failure Assessment score and status of the underlying malignancy at ICU admission were significantly associated with MCS impairment (− 0.54 points [95% CI − 0.99; − 0.1], *p* = 0.018 and − 4.83 points [95% CI − 8.44; − 1.22], *p* = 0.009, respectively).

**Conclusion:**

HRQOL is strongly impaired in critically ill patients with HMs at 3 months and 1 year after ICU discharge. Organ failure and disease status are strongly associated with QOL. The kinetic evaluation of QOL at 3 months and 1 year offers the opportunity to focus on QOL aspects that may be improved by therapeutic interventions during the first year after ICU discharge.

**Electronic supplementary material:**

The online version of this article (10.1186/s13613-018-0478-3) contains supplementary material, which is available to authorized users.

## Background

Over the last few decades, the increased intensity of treatments in patients with haematological malignancies (HMs) has translated into an improved survival rate. As a consequence of more aggressive chemotherapies and their complications, the need for patients with HMs for intensive care unit (ICU) support during the course of their disease has increased. Although outcomes of these patients have been fully investigated in terms of organ failure and mortality [1], data are scarce on health-related quality of life (HRQOL) in this population [2]. Long-term outcomes for physical and psychological factors, functional status and social interactions are becoming more and more important both for physicians and nurses and for patients and their relatives. Moreover, quality-of-life (QOL) measurements should be considered when making decisions on the allocation of healthcare resources.

In the ICU, HRQOL at admission has been shown to be inversely associated with multiple organ failure during hospitalization [3] and hospital mortality [4]. Moreover, baseline HRQOL has been shown to be correlated with HRQOL following discharge [5, 6]. Finally, ICU survivors have significantly lower QOL compared with gender- and age-matched general population [7]. Data on HRQOL in critically ill patients with HMs are scarce [2]. Given the importance of the underlying disease as a strong predictor of HRQOL, the present study focused on critically ill haematological patients. We aim to assess post-ICU burden and HRQOL at 3 months and 1 year after ICU discharge and to identify risk factors for QOL impairment using the three widely used tools in this context: Short Form 36 (SF-36) [8, 9], Hospital Anxiety and Depression Scale (HADS) [10] and Impact of Event Scale (IES) [11].

## Patients and methods

### Description

The study was approved by the appropriate ethics committees in France and Belgium. All patients or relatives were informed and consented to participate in the study. The TRIALOH study was carried out in 17 university or university-affiliated centres in France and Belgium that belonged to a research network instituted in 2005 [1]. From 1 January 2010, to 1 May 2011, consecutive patients with HMs who were admitted to the participating ICUs for any reason were included.

Exclusion criteria were: complete cure of the malignancy for more than 5 years, ICU admission only to maximize safety of a procedure and age younger than 18 years. In each centre, we reported the ICU refusal rate (number of patients considered for ICU admission but eventually not admitted/number of patients admitted to the ICU throughout the study period).

### Data collection

In each centre, a standardized electronic case-report form was used to collect the study data. All QOL-related data were collected prospectively. Ninety days and 1 year after ICU discharge, HRQOL was assessed by asking alive patients to complete the SF-36 questionnaire (SF-36) during a telephone interview by a trained social worker.

The SF-36 includes one multi-item scale that assesses eight health concepts: (1) limitations in physical activities because of health problems; (2) limitations in social activities because of physical or emotional problems; (3) limitations in usual role activities because of physical health problems; (4) bodily pain; (5) general mental health (psychological distress and well-being); (6) limitations in usual role activities because of emotional problems; (7) vitality (energy and fatigue); and (8) general health perceptions [12]. Higher scores represent better functioning, with a range from 0 to 100. Physical and mental summary components (PCS and MCS, respectively) constitute aggregates of the eight individual dimensions and provide norm-based summary scores from physically and mentally oriented subscales. IES and HAD scale (HADS) are self-report instruments that assess the essential characteristics associated with stress and anxiety disorders among patients in a non-psychiatric hospital. The HADS is a 14-item self-report measure of psychological distress. The HADS has two subscales (anxiety and depression), each ranging from 0 to 21. Each item is rated on a scale from 0 (“not at all”) to 3 (“very much”), and higher scores indicate greater anxiety and depression. Scores from the two subscales are combined into a full-scale score that is indicative of a clinical disorder if greater than or equal to 12 points [13]. The IES is a widely used self-report measure of traumatic stress based on two subscales, evaluating intrusion and avoidance. Score of 33 points or more is described as a cut-off for a probable diagnosis of post-traumatic syndrome disorder (PTSD) [14].

### Definitions

Malignancies were considered as newly diagnosed if they had been diagnosed within the past 4 weeks before ICU admission. The sepsis-related organ failure assessment (SOFA) score was computed at admission then daily throughout the patient’s stay in the ICU; this score provides an estimate of the risk of ICU death based on organ dysfunction [15]. Performance status was evaluated using the Eastern Cooperative Oncology Group scale (ECOG). Fully active, able to carry on all pre-disease performance without restriction; restricted in physically strenuous activity but ambulatory and able to carry out work of a light or sedentary nature, e.g. light house work, office work; ambulatory and capable of all self-care but unable to carry out any work activities; up and about more than 50% of waking hours; capable of only limited self-care; confined to bed or chair more than 50% of waking hours; completely disabled; cannot carry on any self-care; totally confined to bed or chair; dead. The ECOG performance status and Charlson comorbidity index were determined at ICU admission [16, 17].

Reasons for ICU admission were recorded based on the main symptoms at ICU admission. Aetiologic diagnoses of sepsis and acute respiratory failure were made by consensus by the intensivists, hematologists and consultants. In particular, aetiologies of pulmonary involvement were diagnosed based on predefined criteria [1, 18].

### Statistical analysis

Continuous variables are described as medians and interquartile ranges (IQRs) and were compared between groups using the Wilcoxon’s rank-sum test. Discrete variables are described with counts and percentages and were compared using Fisher’s exact test. We report norm-based measurements of the SF-36 subscores (mean 50, SD 10), after age and sex standardization using reference values for the French population [19]. SF-36 aggregate components (i.e. PCS and MCS) were computed as recommended [8, 20] and expressed on a normalized scale centred on 50 representing the population norm (i.e. values > or < 50 reflecting values higher or lower than age–sex standardized French values). Missing data in the individual SF-36 questions were imputed using the personal mean score approach [21]. The SF-36 aggregate scores (PCS and MCS) were compared using Wilcoxon’s rank-sum test among groups defined by categorical factors in univariate analysis. Correlations between SF-36 aggregate scores and quantitative variables were estimated and tested using Spearman correlation coefficient (with its 95% confidence interval [95% CI], estimated by bootstrap with 1000 replications). Adjusted multivariate models of factors associated with PCS and MCS were selected, using linear regression models: characteristics available at ICU admission associated with *p* values less than 0.2 by univariate analysis were candidates, and final models were selected using backward stepwise procedures (based on a *p* value < 0.20).

SF-36 scores at 3 months versus 1 year after discharge from the ICU were compared using Wilcoxon’s signed-rank test. Factors associated with the variation of SF-36 were examined using Wilcoxon and Kruskal–Wallis tests (discrete variables), or using Spearman correlation coefficient testing (continuous variables). Last, the age–sex standardized norm-based SF-36 items and aggregate scores in the HM cohort were compared to those of a sepsis cohort of 37 patients, including adult (≥ 18 years old) patients surviving to an ICU hospitalization for a septic shock without HM. These patients were recruited from two prospective studies on sepsis [22, 23]. Secondarily, the comparison was performed after matching patients on age and sex. Factors associated with the IES and HADS scores were investigated with a similar approach, in univariate analysis.

All tests were two-sided, and *p* values less than .05 were considered significant. Analyses were performed using R software version 3.2.2 [24].

## Results

### Patients

**Fig. 1:**
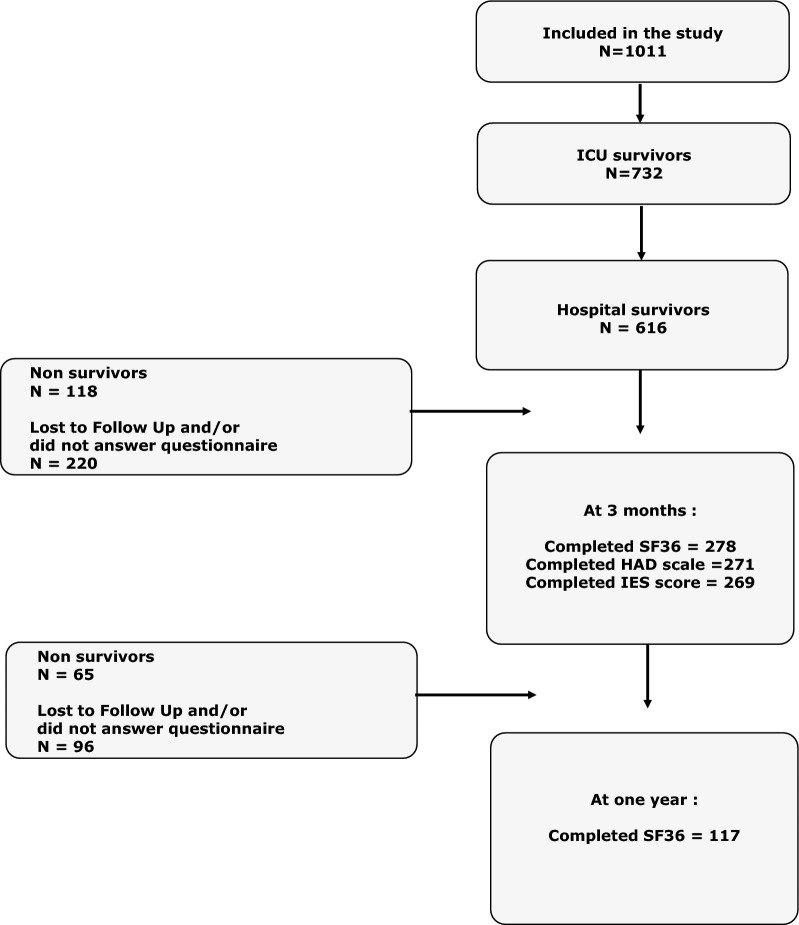
Flowchart of the patient cohort over the 1 year period following intensive care unit (ICU) discharge

In total, 1011 patients were included in the TRIALOH study. In-hospital mortality rates at 3 months and 1 year were 39.1, 50.7 and 57.2%, respectively. At 3 months, 278, 271 and 269 patients completed the SF-36, HADS and IES forms, respectively (Fig. 1).

Table 1 shows patient characteristics of the ICU survivors. Among them, patients who did not complete the SF-36 form at 3 months were older and more likely to require renal replacement therapy than those who completed the form (*p* = 0.02 and *p* = 0.025, respectively). Conversely, bone marrow transplant recipients were more likely to complete the form at 3 months (*p* = 0.024) and 1 year (*p* = 0.062) (Table 1 and Additional file 1: Table S1). At 1 year, we did not find any other difference between responders and non-responders (Additional file 1: Table S1).

**Table 1:** Patient characteristics of ICU survivors. Comparison between ICU survivors who completed the SF-36 form and those who did not

Variables	Hospital survivors *n* (%) or median (IQR)	Patients who did not complete the SF-36 form at 3 months *n* (%) or median (IQR)	Patients who completed the SF-36 form at 3 months *n* (%) or median (IQR)	*p*
*N*	616	338 (55)	278 (45)	
Male	252 (41)	139 (41)	113 (41)	0.87
Age	59 (47; 68)	60 (47; 71)	58 (47; 65)	0.020
Performance status				
0–2	529 (86)	286 (85)	243 (87)	
3–4	85 (14)	50 (15)	35 (13)	
Charlson index	4 (2;5)	4 (2; 6)	4 (2; 5)	0.024
Malignancies				0.68
AML	162 (26)	82 (24)	80 (29)	
Non-Hodgkin’s lymphoma	191 (31)	111 (33)	80 (29)	
Hodgkin’s lymphoma	18 (3)	9 (3)	9 (3)	
ALL	44 (7)	22 (7)	22 (8)	
CLL	45 (7)	24 (7)	21 (8)	
CML	14 (2)	10 (3)	4 (1)	
Myeloma	86 (14)	51 (15)	35 (13)	
Myelodysplastic syndrome	26 (4)	14 (4)	12 (4)	
BMT/HSCT recipient				0.024
Autologous	78 (13)	37 (11)	41 (15)	
Allogeneic	70 (11)	30 (9)	40 (14)	
Disease status at admission				0.16
Newly diagnosed	237 (41)	135 (42)	102 (39)	
Partial remission	46 (8)	23 (7)	23 (9)	
Complete remission	111 (19)	52 (16)	58 (23)	
SOFA score	5 (3; 7)	5 (3; 7)	5 (3; 7)	0.67
Mechanical ventilation	190 (31)	111 (33)	79 (28)	0.22
Vasopressors	219 (36)	111 (33)	100 (36)	0.93
Renal replacement therapy	106 (18)	68 (21)	38 (14)	0.025
Sedation	177 (29)	100 (30)	77 (28)	0.59
ICU LOS (days)	6 (3;10)	6 (4; 10)	5 (3; 10)	0.24

*MDS* myelodysplastic syndrome, *AML* acute myeloid leukaemia, *ALL* acute lymphocytic leukaemia, *CLL* chronic lymphocytic leukaemia, *CML* chronic myeloid leukaemia, *BMT* bone marrow transplantation, *HSC* haematopoietic stem cell transplant, *SOFA* sepsis-related organ failure assessment, *ICU* intensive care unit, *LOS* length of stay

### SF-36 score at 3 months and 1 year after ICU discharge

**Fig. 2:**
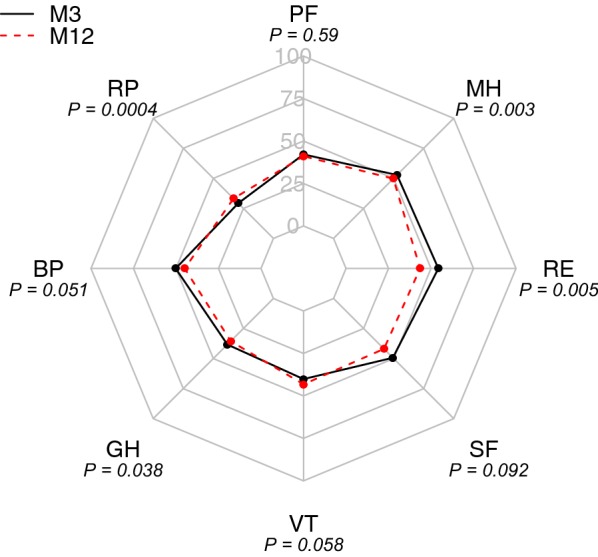
Radar chart of the health-related quality of life (HRQOL) at 3 and 12 months after ICU discharge. M3, 3 months after ICU discharge; M12, 12 months after ICU discharge; RP, role—function physical; PF, physical functioning; MH, mental health; RE, role—emotional; SF, social functioning; VT, vitality; GH, general health; and BP, bodily pain

The results of SF-36 scores at 3 months and 1 year are shown in Fig. 2. At 3 months, the median [IQR] age–sex standardized norm-based physical component summary score (PCS) was 37 [28–46], which corresponds to − 1.3 SD compared to the French general population, and the median [IQR] mental component summary score (MCS) was 51 [45–58]. Median age–sex standardized MCS values were similar (0.1 SD) to those of the general population in HMs patients (Table 2).

**Table 2:** SF-36 score evolution from 3 months to 1 year after ICU discharge

Scores	Median (IQR) at 3 months age–sex standardized	Median variation (IQR) after 1 year evolution	*p*
Physical score component	37 (28; 46)	0.0 (− 5.2; 6.5)	0.38
Mental score component	51 (45; 58)	− 2.9 (− 10.7; 5.3)	0.006
Physical functioning (PF)	40 (28; 51)	0.0 (− 9.2; 8.0)	0.59
Role—physical (RP)	32 (26; 41)	0.0 (0.0; 15.1)	0.0004
Bodily pain (BP)	50 (38; 62)	− 2.1 (− 10.2; 5.6)	0.051
General health (GH)	38 (30; 48)	− 2.7 (− 12.6; 5.3)	0.038
Vitality (VT)	41 (31; 48)	2.7 (− 5.7; 11.0)	0.058
Social functioning (SF)	51 (31; 58)	0.0 (− 15.6; 10.7)	0.092
Role—emotional (RE)	54 (38; 55)	0.0 (− 19.1; 0.0)	0.005
Mental health (MH)	53 (45; 60)	− 2.4 (− 9.6; 2.3)	0.003

All subscores were not affected proportionally: while the median role function—physical (RP), median vitality (VT) and median general health (GH) scores were 32 [26–41], 41 [31–48] and 38 [30–48], respectively, median role function—emotional (RE) score, median mental health (MH), median social functioning (SF) and bodily pain (BP) scores were 54 [38–55], 53 [45–60], 51 [31–58] and 50 [38–62], respectively.

Evolution of these scores over time is shown in Fig. 2 and Table 2. MH, RE and GH scores decreased significantly between 3 months and 1 year after ICU discharge (*p* = 0.003, *p* = 0.005 and *p* = 0.038, respectively). MCS decreased significantly between 3 months and 1 year (*p* = 0.006). Conversely, RP increased significantly between 3 months and 1 year (*p* = 0.0004). Performance status has been shown to be strongly correlated with physical role functioning and functional capacity [25, 26]. When considering the performance status as a marker of functional capacity at baseline, we found a decrease in functional capacity from ICU admission to 3 months, with partial recovery in survivors at 1 year (Additional file 1: Figure S1).

**Fig. 3:**
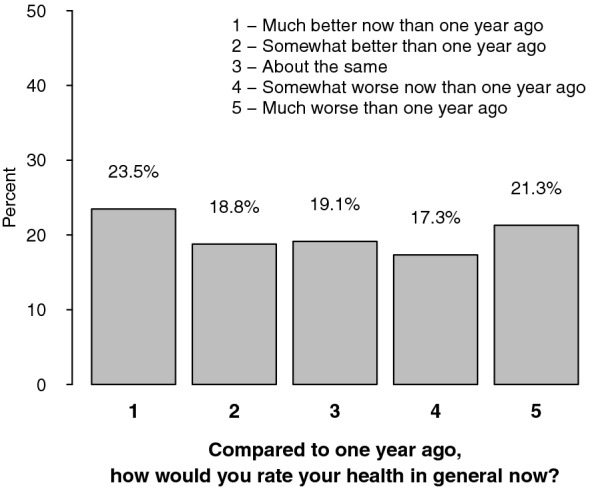
Item 2 of the SF-36 “Compared to 1 year ago, how would you rate your health in general now?”

Detailed answers to the question “Compared to 1 year ago, how would you rate your health in general now?” (item 2 of the SF-36) are shown in Fig. 3. Among survivors, 42% of patients felt better than 1 year before and 38.6% felt worse than 1 year before.

### Comparison of critically ill patients with HMs with septic shock patients without HMs

We then compared QOL 3 months after discharge in critically ill patients with HMs with QOL in critically ill patients with septic shock without HMs. After matching for age and sex, we found that median PCS values were higher in septic shock patients than in HMs patients (52 [37–59] vs. 37 [29–46], *p* = 0.007, respectively). There was no significant difference between HMs and septic shock survivors regarding the MCS aggregate of the SF-36 questionnaire (54 [45–58] vs. 50 [45–58], *p* = 0.99, respectively) (Table 3). PF, RP, VT and SF subscores were significantly higher in septic shock patients than in HMs patients (*p* = 0.016, *p* = 0.007, *p* = 0.035 and *p* = 0.007, Table 3).

**Table 3:** Components of the Medical Outcome Study SF-36 questionnaire for critically ill patients with haematological malignancies (HMs) and septic shock patients without HM

	HM median (IQR) age–sex standardized^a^ SD^b^		Septic shock median (IQR) age–sex standardized^a^ SD^b^		*p*
*N*	37		37		
Age	52 (37; 64)		53 (37; 62)		
Sex (female)	23 (62)		23 (62)		
Physical functioning (PF)	43 (30; 54)	− 0.7 (− 2.0; 0.4)	50 (43; 56)	0.0 (− 0.7; 0.6)	0.015
Role—physical (RP)	34 (26; 41)	− 1.6 (− 2.4; − 0.9)	44 (32; 54)	− 0.6 (− 1.8; 0.4)	0.007
Bodily pain (BP)	47 (37; 60)	− 0.3 (− 1.3; 1.0)	57 (39; 60)	0.7 (− 1.1; 1.0)	0.34
General health (GH)	41 (33; 50)	− 0.9 (− 1.7; 0.0)	41 (30; 56)	− 0.9 (− 2.0; 0.6)	0.84
Vitality (VT)	41 (29; 48)	− 0.9 (− 2.1; − 0.2)	48 (39; 53)	− 0.2 (− 1.1; 0.3)	0.035
Social functioning (SF)	44 (28; 58)	− 0.6 (− 2.2; 0.8)	54 (42; 58)	0.4 (− 0.8; 0.8)	0.042
Role—emotional (RE)	47 (37; 55)	− 0.3 (− 1.3; 0.5)	54 (41; 55)	0.4 (− 0.9; 0.5)	0.48
Mental health (MH)	54 (50; 60)	0.4 (− 0.0; 1.0)	53 (45; 58)	0.3 (− 0.5; 0.8)	0.21
Physical Component Summary (PCS)	37 (29; 46)	− 1.3 (− 2.1; − 0.4)	52 (37; 59)	0.2 (− 1.3; 0.9)	0.007
Mental Component Summary (MCS)	50 (45; 58)	− 0.0 (− 0.5; 0.8)	54 (45; 58)	0.4 (− 0.5; 0.8)	0.99

^a^SF36 values are centred around 50 (i.e. values > or < 50 reflect values higher or lower than age–sex standardized values in the general French population)

^b^SF36 values are centred around 0 (i.e. values > or < 0 reflect values higher or lower than age–sex standardized French values in the general French population, in standard deviation unit)

### Factors associated with QOL

**Fig. 4:**
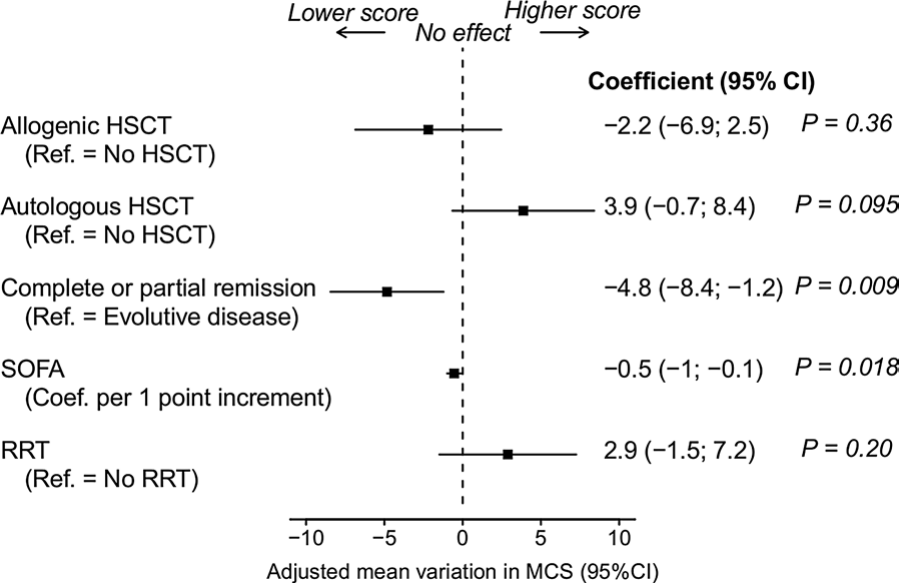
Multivariate analysis: effects on mental component summary score of covariates identified by multivariate linear regression

Overall, 3 months after ICU discharge, the need for invasive mechanical ventilation (MV), baseline status of the underlying malignancy (partial or complete remission of the disease vs. evolutive), the need for renal replacement therapy, the need for vasopressors, sedation, SOFA score and haematopoietic stem cell transplant were associated with the MCS at a 20% level in univariate analysis (Additional file 1: Table S2); they were included as candidate factors in a multivariate adjusted model. After selection, SOFA score and baseline status of the underlying malignancy remained independently associated with MCS impairment (− 0.54 points [95% CI − 0.99; − 0.1], *p* = 0.018 and − 4.83 points [95% CI − 8.44; − 1.22], *p* = 0.009, respectively) (Fig. 4).

The only variable associated with PCS impairment at 3 months at a 20% level in univariate analysis was the Charlson index (Additional file 1: Table S3).

Of note, patients in partial or complete remission had a longer median time from the diagnosis of the underlying malignancy and ICU admission than patients classified as having an evolutive disease. Indeed, most patients in the latter case (57.3% of the patients classified as having an evolutive disease) had a newly diagnosed malignancy when admitted to the ICU (Additional file 1: Figure S2)

We did not find any association between these variables and MCS or PCS at 1 year after ICU discharge. (Univariate analyses are shown in Additional file 1: Tables S4 and S5.)

### Post-traumatic stress disorder

Three months after ICU discharge, 269 patients completed the IES form. The median IES score was 8 [3, 16]. The median intrusion subscore was 3 [0; 9], and the median avoidance subscore was 3 [0; 8]. Overall, 22 (8%) patients had an IES score greater than 35 points, which is considered the threshold for PTSD.

Men had a higher IES score than women (10 [4–21] vs. 6 [1–13], respectively, *p* = 0.0008). The IES score was positively correlated with length of stay in the ICU (correlation coefficient 0.14 [95% CI 0.02; 0.026], *p* = 0.025) (Additional file 1: Figure S3). By univariate analysis, ICU length of stay was associated with IES score at 3 months (Additional file 1: Table S6).

### Anxiety and depression

Three months after ICU discharge, patients had a median combined HAD score of 8 [5–13]. Considering both the subscales separately, 42 patients (15.5%) had an anxiety score > 8 and 41 patients (15%) had a depression score > 8, which are considered clinical disorders.

## Discussion

This prospective study is the first multicenter study and the largest one focusing on HRQOL in critically ill patients with HMs.

To analyse all aspects of QOL, we used the SF-36 form, which offers a detailed mental and physical QOL evaluation and has been widely used and validated in ICU patients, the IES for post-traumatic stress syndrome evaluation and the HADS for evaluation of anxiety and depression.

In total, 1011 patients were included in the TRIALOH study. With a mortality rate of 50.7% (513 patients) at 3 months, 55.8% of survivors (278 patients) completed the SF-36 form at 3 months. Although response rates vary a lot between studies in ICU, ranging from 30 to 80% [27, 28], our response rate is somewhat low (56% of the survivors) and may be explained in part by the high morbidity of patients with HMs, who require multiple hospitalizations and treatments in the course of their disease. Indeed, more days spent at hospital have been associated with no response at follow-up [28]. Populations who are lost to follow-up or did not answer the questionnaire may have different demographic characteristics and different profiles of ICU-related morbidity than those who completed the QOL evaluation. Indeed, we found that age and the need for replacement therapy during ICU stay were 2 factors associated with the inability/refusal to complete the form. Different methods have been used during the study to improve response rates, such as sending hand written letters and employing reminders to the patients.

Compared with general ICU patients with septic shock, critically ill patients with HMs have profound alterations of HRQOL at 3 months. Moreover, although RP values improved at 1 year, global QOL impairment was consolidated at 1 year.

In a single-centre study, Oeyen et al. [2] investigated long-term outcomes and QOL in critically ill patients with HMs (85 patients) or solid malignancies 3 months and 1 year after ICU discharge. Similarly, they found a profound alteration of QOL at 3 months and 1 year in HM patients with median PCS and MCS values even lower than those we observed in our study. Practices may have evolved over time, taking into account the QOL as an important goal when managing these patients. This may partly explain the discrepancy between the two studies.

In the present study, two main independent factors were significantly associated with QOL impairment (MCS) at 3 months: the SOFA score (i.e. organ failure) and the status of the underlying malignancy (complete or partial remission) at ICU admission. The initial SOFA score is used to quantify the degree of organ dysfunction present on admission, including the need for mechanical ventilation, vasopressors or renal replacement therapy. Many studies have demonstrated a strong correlation of initial SOFA score with mortality outcome [15, 29, 30]. In our study, we have moved a step further, presenting SOFA score as a reliable predictor of QOL after ICU discharge. Combes et al. [31] previously showed that prolonged invasive mechanical ventilation is associated with impaired HRQOL compared with that of a matched general population. Noninvasive strategies during ICU stay, such as decreasing the use of mechanical ventilation by using high flow oxygen, when appropriate, may improve QOL.

SOFA score has previously been associated with impaired QOL in critically ill patients with acute respiratory distress syndrome [32]. In clinical practice, a crucial question is “for which critically ill HMs patients should we propose a limitation or withdrawal of life-sustaining treatments”? Decisions on withholding/withdrawing therapies should take into account the expected QOL after ICU discharge; the primary goal of intensive care being to return to a quality of life the patient would have found acceptable. Considering the SOFA score at admission as a predictor of both mortality and QOL, it may be helpful to physicians to inform patients and families in a reliable way and to guide in treatment decisions.

Paradoxically, disease status (partial or complete remission) at ICU admission was also associated with MCS impairment at 3 months. Patients in partial or complete remission have previously received intensive and potentially gruelling treatments for their underlying malignancy; conversely, most of patients classified as having an “evolutive disease” have newly diagnosed malignancies when admitted to the ICU. Indeed, patients who are considered in partial or complete remission had a longer time from the diagnosis of the underlying malignancy to ICU admission and, overall, have therefore received more chemotherapy than patients with evolutive disease.

The kinetic evaluation of QOL at 3 months and 1 year offers the opportunity to focus on aspects of QOL that may be improved by therapeutic interventions during the first year after ICU discharge. If some aspects of QOL (such as RP) have improved over time, we found that BP, GH, MH, RE scores and MCS decreased between 3 months and 1 year after ICU discharge. In general ICU populations, previous studies have shown that the majority of the QOL scores recover over time [5]. Impairment of SF and RE suggests that patients with HMs may be socially isolated because of their condition. Association of a long hospital stay, including ICU stay and the presence of an underlying malignancy, has been shown to be major risk factors for social isolation [33, 34].

During hospitalization, HM patients have to deal not only with treatment-related complications and adverse events, such as physical symptoms and changes in body image, but also with isolation-related psychological distress, including loss of control and lack of contact with family members and friends. Long-term psychological follow-up, social support and recreational activities during hospitalization may improve the QOL of these patients [34, 35]. Centres that manage haematological patients may also consider extending visiting hours for relatives. Finally, bodily pain may be controlled by an approach carried out by a skilled pain care team and based on the association of causal therapies and adequate analgesics. PF, RP, VT and PCS are significantly lower in HMs patients compared with septic patients. Indeed, HMs and chemotherapies have been shown to induce a loss of weight and decrease in physical activity. Early mobilization in ICU and exercise may prevent the rapid loss of physical reserve and increase functional capacity [36].

In the princeps study published in 2013 by Azoulay et al. [1], 6 months after ICU discharge, the hematologists reported that all but seven ICU survivors were continuing their cancer treatment that ICU admission did not influence therapeutic intensity in 80% of ICU survivors, and that 80% of ICU survivors were in complete or partial remission. Unfortunately, precise data on post-ICU treatments are not available and we could not evaluate their impact on QOL.

Interestingly, only 8% of patients in our cohort were diagnosed with PTSD, according to the IES, which is lower than usually reported [37]. The use of an ICU diary in many participating centres may have participated in the low incidence of PTSD as an ICU diary has been associated with a significant reduction in PTSD symptoms in critical illness survivors [37]. A prospective multicenter comparative study of the impact of an ICU diary on PTSD in ICU is ongoing, involving many of the participating centres involved in this work.

The present study has several limitations. First, we were unable to provide QOL measures at ICU admission. Emergent admission and critical illness prevent patients from providing self-reported baseline QOL at the time of ICU admission. Reliable retrospective QOL data are difficult to obtain due to memory biases following ICU discharge. Answers to the question “Compared to 1 year ago, how would you rate your health in general now?” suggest that almost half of the patients felt better than 1 year before. Factors such as severity of current symptoms may bias patients’ recall of baseline status, especially in more severe patients, such as ICU patients [38]. Indeed, Granja et al. [39] have shown that almost 50% of ICU survivors did not remember the time in the hospital before ICU admission. Moreover, proxy assessment of patients’ baseline quality of life often differs from the patient’s assessment [40]. Despite these limitations, HRQOL impairment at ICU admission has been correlated with multiple organ failure during ICU stay, increased hospital mortality and worsened HRQOL following discharge [4, 6].

Second, as mentioned above, the response rate (55.8% of the survivors) is low, which is explained by the high morbidity of our population.

Third, our data are now 7 years old. Improvements in outcomes of cancer patients, as well as the improvements in process of care may have modified the impact of ICU complications on HRQOL. In HM patients, several cohort studies and trials evaluating a noninvasive diagnostic and therapeutic management of acute respiratory failure have shown the feasibility and safety of the noninvasive strategy [41]. High flow oxygen has also demonstrated survival benefits as compared to noninvasive ventilation [42]. The increased use of noninvasive strategies may then have improved HRQOL of these patients after ICU discharge.

Finally, a longer follow-up would have been interesting to analyse, as a gradual improvement of most aspects of QOL might have occurred after 1 year.

## Conclusion

This study shows that HRQOL is strongly impaired in critically ill patients with HMs at 3 months and 1 year after ICU discharge. SOFA score and disease status are strongly correlated with QOL. Recovery after the ICU is not consistent in all dimensions of HRQOL. Future studies should focus on specific physical and psychosocial rehabilitation programs that may start in the ICU. They may then continue after ICU discharge and could lead to improved management of patients with HMs after their stay in the ICU.

## Additional file

**Additional file 1.** Tables and figures.

## Abbreviations

ICU: intensive care unit
HM: haematological malignancy
HRQOL: health-related quality of life
QOL: quality of life
SF-36: Short Form 36 questionnaire
HADS: Hospitality and Depression Scale
IES: Impact of Event Scale
PTSD: post-traumatic stress disorder
SOFA: the sepsis-related organ failure assessment
MDS: myelodysplastic syndrome
AML: acute myeloid leukaemia
CLL: chronic lymphocytic leukaemia
CML: chronic myeloid leukaemia
BMT: bone marrow transplantation
HSCT: haematopoietic stem cell transplant
LOS: length of stay
ALL: acute lymphoid leukaemia
MCS: mental component summary score
PCS: physical component summary score
RP: role function—physical
VT: vitality
MH: mental health
GH: general health
RE: role—emotional
BP: bodily pain
SF: social functioning
PF: physical functioning
