# Supplementary material for: Long-term health-related quality of life of critically ill patients with haematological malignancies: a prospective observational multicenter study
Source: Ann Intensive Care. 2019 Jan 5;9:2. doi: 10.1186/s13613-018-0478-3 (PMC6320707; doi:10.1186/s13613-018-0478-3)
Supplement: Supplementary file 1 — Additional file 1. Tables and figures. [file 13613_2018_478_MOESM1_ESM.docx]

**Supplemental data**

| Variables | Patients who did not complete the SF-36 form at 1 year  n (%) or median (IQR) | Patients who completed the SF-36 form at 1 year  n (%) or median (IQR) | P |
| --- | --- | --- | --- |
| N | 161 (58) | 117 (42) |  |
| Male | 59 (37) | 54 (46) | 0.14 |
| Age | 58 (47 ;66) | 57 (47 ;64) | 0.44 |
| Performance Status |  |  | 1 |
| 0-2 | 141 (88) | 102 (87) |  |
| 3-4 | 20 (12) | 15 (13) |  |
| Charlson index | 4 (2 ;5) | 4 (2 ;5) | 0.25 |
| Malignancies |  |  | 0.32 |
| AML | 49 (30) | 31 (26) |  |
| Non Hodgkin’s lymphoma | 51 (32) | 29 (25) |  |
| Hodgkin’s lymphoma | 3 (2) | 6 (5) |  |
| ALL | 10 (6) | 12 (10) |  |
| CLL | 9 (6) | 12 (10) |  |
| CML | 3 (2) | 1 (1) |  |
| Myeloma | 19 (12) | 16 (14) |  |
| Myelodysplastic syndrome | 9 (6) | 3 (3) |  |
| BMT/HSCT recipient |  |  | 0.062 |
| No graft | 121 (74) | 75 (65) |  |
| Autologous | 17 (11) | 24 (21) |  |
| Allogenic | 23 (14) | 17 (15) |  |
| Disease status at admission |  |  | 0.74 |
| Newly diagnosed | 58 (37) | 44 (42) |  |
| Partial remission | 16 (10) | 7 (7) |  |
| Complete remission | 36 (23) | 23 (22) |  |
| SOFA score | 5 (3 ;8) | 5 (4 ;7) | 0.67 |
| Mechanical ventilation | 45 (28) | 34(29) | 0.89 |
| Vasopressors | 63 (39) | 37 (32) | 0.21 |
| Renal replacement therapy | 20 (12) | 18 (16) | 0.48 |
| Sedation | 44 (27) | 33 (28) | 0.89 |
| ICU LOS (days) | 5 (3 ;9) | 6 (3 ;11) | 0.37 |

**Table S1**. Comparaison between ICU survivors who completed the SF36 form at 1 year and those who did not. MDS, myelodysplastic syndrome; AML, acute myeloid leukaemia ; ALL, acute lymphocytic leukaemia ; CLL, chronic lymphocytic leukaemia; CML, chronic myeloid leukaemia; BMT, bone marrow transplantation; HSC, haematopoietic stem-cell transplant; SOFA, sepsis-related organ failure assessment; ICU, intensive care unit; LOS, length of stay.

| Variables | N (%) | Median (IQR)  or Spearman (95%) | Age-sex standardized values  (SD) | | P |
| --- | --- | --- | --- | --- | --- |
| Gender |  |  |  | |  |
| Male | 111 (41) | 53.9 (47.2;59.7) |  |  | |
| Female | 160 (59) | 49.8 (42.0;56.9) |  | |  |
| Age | 271 | 0.01 (-0.1;0.13) |  | |  |
| Performance Status |  |  |  | | 0.23 |
| 0-2 | 237 (87) | 50.8 (44.6;58.2) | 0.08 (-0.54;0.82) | |  |
| 3-4 | 34 (13) | 55.3 (46.2;61.2) | 0.53 (-0.38;1.12) | |  |
| Charlson | 270 | -0.04 (-0.15;0.09) | -0.04 (-0.15;0.09) | | 0.56 |
| Malignancies |  |  |  | | 0.51 |
| NHL / Hodgkin | 86 (32) | 52.3 (42.6;60.2) | 0.23 (-0.74;1.02) | |  |
| ALL / AML | 101 (37) | 50.2 (46.4;57.5) | 0.02 (-0.36;0.75) | |  |
| CLL / others | 34 (13) | 50.8 (44.2;56.9) | 0.08 (-0.58;0.69) | |  |
| CML / MDS | 16 (6) | 48.5 (42.3;56.8) | -0.15 (-0.77;0.68) | |  |
| Myeloma | 34 (13) | 54.8 (46.8;59.4) | 0.48 (-0.32;0.94) | |  |
| BMT / HSCT recipient |  |  |  | | 0.024 |
| No graft | 190 (70) | 51.5 (46.1;59.5) | 0.15 (-0.39;0.95) | |  |
| Autologous | 41 (15) | 47.6 (39.4;52.1) | 0.33 (-0.83;0.88) | |  |
| Allogenic | 39 (14) | 53.3 (41.7;58.8) | -0.24 (-1.06;0.21) | |  |
| Disease status at admission |  |  |  | | 0.0002 |
| Newly diagnosed | 173 (68) | 53.3 (47.5;60.2) | 0.33 (-0.25;1.02) | |  |
| Partial or complete remission | 80 (32) | 48.2 (38.8;54.6) | -0.18 (-1.12;0.46) | |  |
| SOFA score | 227 | -0.1 (-0.24;0.03) | -0.1 (-0.24;0.03) | | 0.12 |
| Mechanical Ventilation |  |  |  | | 0.16 |
| Yes | 75 (28) | 49.2 (41.8;58.1) | -0.08 (-0.82;0.81) | |  |
| No | 196 (72) | 51.6 (46.0;58.4) | 0.16 (-0.40;0.84) | |  |
| Vasopressors |  |  |  | | 0 .0064 |
| Yes | 98 (36) | 49.3 (42.7;56.7) | -0.07 (-0.73;0.67) | |  |
| No | 173 (64) | 51.8 (46.3;59.4) | 0.18 (-0.37;0.94) | |  |
| Renal replacement therapy |  |  |  | | 0.11 |
| Yes | 37 (14) | 53.0 (47.7;59.9) | 0.30 (-0.23;0.99) | |  |
| No | 230 (86) | 50.3 (43.9;58.1) | 0.03 (-0.61;0.81) | |  |
| Sedation |  |  |  | |  |
| Yes | 74 (27) | 48.3 (41.7;58.0) | -0.17 (-0.83;0.80) | | 0.06 |
| No | 197 (73) | 51.6 (46.3;58.4) | 0.16 (-0.37;0.84) | |  |
| ICU LOS (days) | 271 | 0.02 (-0.1;0.13) | 0.02 (-0.1;0.13) | | 0.80 |

Table S2. Association between different variables and age-sex standardized mental component summary score (MCS) 3 months after ICU discharge by univariate analysis.

| Variables | N (%) | Median (IQR)  or Spearman (IC 95%) | Age-sex standardized values  (SD) | P |
| --- | --- | --- | --- | --- |
| Gender |  |  |  |  |
| Male | 111 (41) | 35.7 (27.1;45.0) |  |  |
| Female | 160 (59) | 38.5 (29.3;48.3) |  |  |
| Age | 271 | 0.3 (0.18;0.42) |  |  |
| Performance Status |  |  |  | 0.28 |
| 0-2 | 237 (87) | 36.9 (28.1;46.9) | 0.08 (-0.54;0.82) |  |
| 3-4 | 34 (13) | 32.7 (27.4;41.7) | 0.53 (-0.38;1.12) |  |
| Charlson | 270 | 0.15 (0.03;0.27) | -0.04 (-0.15;0.09) | 0.013 |
| Malignancies |  |  |  | 0.28 |
| NHL / Hodgkin | 86 (32) | 36.3 (25.4;45.0) | 0.23 (-0.74;1.02) |  |
| ALL / AML | 102 (37) | 36.5 (27.8;44.6) | 0.02 (-0.36;0.75) |  |
| CLL / others | 36 (13) | 43.3 (31.4;48.9) | 0.08 (-0.58;0.69) |  |
| CML / MDS | 16 (6) | 33.5 (28.5;44.8) | -0.15 (-0.77;0.68) |  |
| Myeloma | 35 (13) | 36.1 (29.8;49.3) | 0.48 (-0.32;0.94) |  |
| BMT / HSCT recipient |  |  |  | 0.90 |
| No graft | 190 (70) | 36.5 (28.0;45.9) | 0.15 (-0.39;0.95) |  |
| Autologous | 41 (15) | 37.9 (30.8;45.0) | 0.33 (-0.83;0.88) |  |
| Allogenic | 39 (14) | 34.6 (24.1;47.6) | -0.24 (-1.06;0.21) |  |
| Disease status at admission |  |  |  | 0.41 |
| Newly diagnosed | 173 (68) | 36.7 (29.2;47.0) | 0.33 (-0.25;1.02) |  |
| Partial or complete remission | 82 (32) | 34.3 (25.3;45.4) | -0.18 (-1.12;0.46) |  |
| SOFA score | 227 | 0.04 (-0.09;0.17) | -0.1 (-0.24;0.03) | 0.50 |
| Mechanical Ventilation |  |  |  | 0.27 |
| Yes | 75 (28) | 34.3 (27.0;44.6) | -0.08 (-0.82;0.81) |  |
| No | 196 (72) | 37.1 (28.5;47.1) | 0.16 (-0.40;0.84) |  |
| Vasopressors |  |  |  | 0.92 |
| Yes | 98 (36) | 35.6 (26.9;47.0) | -0.07 (-0.73;0.67) |  |
| No | 173 (64) | 36.7 (28.6;45.6) | 0.18 (-0.37;0.94) |  |
| Renal replacement therapy |  |  |  | 0.26 |
| Yes | 38 (14) | 33.6 (25.9;43.9) | 0.30 (-0.23;0.99) |  |
| No | 230 (86) | 37.1 (28.7;46.9) | 0.03 (-0.61;0.81) |  |
| Sedation |  |  |  | 0.34 |
| Yes | 74 (27) | 36.9 (28.6 ; 47.0) | -0.17 (-0.83;0.80) |  |
| No | 197 (73) | 33.3 (26.9;44.9) | 0.16 (-0.37;0.84) |  |
| ICU LOS (days) | 271 | -0.06(-0.19;0.06) | 0.02 (-0.1;0.13) | 0.33 |

Table S3. Association between different variables and age-sex standardized physical summary component score (PCS) 3 months after ICU discharge by univariate analysis.

Figure S1. Evolution of functional capacity over time. RP: Part 4 of SF36 questionnaire, dichotomized according to the number of declared limitations in activities (number of “yes” answers to the four questions: 0-2 (better) vs. 3-4 (worse)). PS: Performance Status


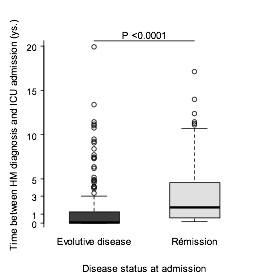


### Figure S2 Time between ICU admission and diagnosis of the underlying malignancy according to disease status.

| Variables | N (%) | Median (IQR) or Spearman (95%) | P |
| --- | --- | --- | --- |
| Gender |  |  |  |
| Male | 62 (54) | 45.3 (36.5;53.0) |  |
| Female | 52 (46) | 49.5 (43.5;56.4) |  |
| Age | 114 | -0.04 (-0.22;0.14) |  |
| Performance Status |  |  | 0.39 |
| 0-2 | 99 (87) | 48.6 (38.2;55.0) |  |
| 3-4 | 15 (13) | 50.4 (47.1;54.6) |  |
| Charlson | 113 | -0.01 (-0.18;0.16) | 0.93 |
| Malignancies |  |  | 0.044 |
| NHL / Hodgkin | 34 (30) | 45.6 (38.4;50.8) |  |
| ALL / AML | 41 (36) | 47.4 (37.9;53.9) |  |
| CLL / others | 19 (17) | 54.0 (41.2;57.0) |  |
| CML / MDS | 4 (4) | 39.5 (33.2;46.5) |  |
| Myeloma | 16 (14) | 52.2 (48.1;58.8) |  |
| BMT / HSCT recipient |  |  | 0.65 |
| Autologous | 24 (21) | 47.1 (42.1;51.2) |  |
| Allogenic | 17 (15) | 43.0 (35.4;57.5) |  |
| Disease status at admission |  |  | 0.056 |
| Newly diagnosed | 72 (71) | 49.2 (42.9;56.2) |  |
| Partial or complete remission | 29 (29) | 47.4 (36.3;50.5) |  |
| SOFA score | 96 | 0.03 (-0.17;0.23) | 0.75 |
| Mechanical ventilation |  |  | 0.50 |
| Yes | 32 (28) | 48.8 (41.7;56.5) |  |
| No | 82 (72) | 48.6 (38.1;54.5) |  |
| Vasopressors |  |  | 0.77 |
| Yes | 36 (32) | 48.9 (42.2;54.7) |  |
| No | 78 (68) | 48.4 (37.9;55.2) |  |
| Renal replacement therapy |  |  | 0.32 |
| Yes | 17 (15) | 49.2 (48.2;53.9) |  |
| No | 94 (85) | 48.0 (37.9;55.1) |  |
| Sedation |  |  | 0.39 |
| Yes | 31 (27) | 48.9 (41.5;56.9) |  |
| No | 83 (73) | 48.2 (38.2;54.4) |  |
| ICU LOS (days) | 114 | 0.02 (-0.16;0.20) | 0.79 |

Table S4. Association between different variables and age-sex standardized mental component summary score (MCS) 1 year after ICU discharge by univariate analysis.

| Variables | N (%) | Median (IQR) or Spearman (95%) | P |
| --- | --- | --- | --- |
| Gender |  |  |  |
| Male | 62 (54) | 36.4 (26.8;43.8) |  |
| Female | 52 (46) | 40.0 (30.6;47.4) |  |
| Age | 114 | 0.32 (0.15;0.46) |  |
| Performance Status |  |  | 0.29 |
| 0-2 | 99 (87) | 37.4 (29.8;47.1) |  |
| 3-4 | 15 (13) | 32.9 (30.9;42.0) |  |
| Charlson | 113 | 0.14 (-0.03;0.3) | 0.14 |
| Malignancies |  |  | 0.28 |
| NHL / Hodgkin | 34 (30) | 34.8 (28.4;43.8) |  |
| ALL / AML | 41 (36) | 37.4 (29.2;47.4) |  |
| CLL / others | 19 (17) | 42.7 (36.1;51.2) |  |
| CML / MDS | 4 (4) | 40.4 (34.3;44.5) |  |
| Myeloma | 16 (14) | 35.7 (30.3;42.1) |  |
| BMT / HSCT recipient |  |  | 0.31 |
| Autologous | 24 (21) | 32.6 (27.5;41.9) |  |
| Allogenic | 16 (14) | 39.7 (30.3;47.4) |  |
| Disease status at admission |  |  | 0.64 |
| Newly diagnosed | 72 (71) | 36.7 (27.6;47.3) |  |
| Partial or complete remission | 29(29) | 41.1 (31.0;43.9) |  |
| SOFA score | 96 | 0.07 (-0.12;0.25) | 0.53 |
| Mechanical Ventilation |  |  | 0.51 |
| Yes | 32 (28) | 36.7 (29.2;43.6) |  |
| No | 82 (72) | 38.3 (30.2;47.1) |  |
| Vasopressors |  |  | 0.80 |
| Yes | 36 (32) | 37.3 (31.5;47.4) |  |
| No | 78 (68) | 36.6 (29.4;45.8) |  |
| Renal replacement therapy |  |  | 0.60 |
| Yes | 17 (15) | 39.6 (31.8;44.0) |  |
| No | 94 (85) | 36.9 (28.3;47.1) |  |
| Sedation |  |  | 0.94 |
| Yes | 31 (27) | 37.4 (30.0;45.6) |  |
| No | 83 (73) | 37.1 (30.1;46.6) |  |
| ICU LOS (days) | 114 | -0.06 (-0.25;0.12) | 0.51 |

Table S5. Association between different variables and age-sex standardized physical component summary score (PCS) 1 year after ICU discharge by univariate analysis.


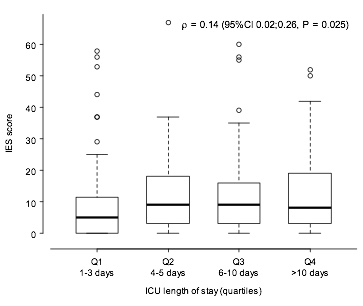


**Figure S3. Boxplot of IES score by quartile of ICU length of stay** (Q1=1^st^ quartile, 1 to 3 days, n=79; Q2=2^nd^ quartile, 4 to 5 days, n=54; Q3=3^rd^ quartile, 6 to 10 days, n=69; Q4=4^th^ quartile, >10 days with maximum of 76 days in our cohort, n=61). Spearman’s rank correlation coefficient (ρ) was estimated, with bootstrapped 95% confidence interval.

| Variables | N (%) | Median (IQR) or Spearman (95%) | P |
| --- | --- | --- | --- |
| Gender |  |  | 0.008 |
| Male | 103 (39) | 10 (4;21) |  |
| Female | 160 (61) | 6 (1;13) |  |
| Age | 263 | -0.07 (-0.19;0.06) | 0.26 |
| Performance Status |  |  | 0.86 |
| 0-2 | 231 (88) | 8 (3;16) |  |
| 3-4 | 32 (12) | 6 (3;15) |  |
| Charlson | 262 | -0.06 (-0.21;0.06) | 0.31 |
| Malignancies |  |  | 0.99 |
| NHL / Hodgkin | 84 (32) | 8 (2;19) |  |
| ALL / AML | 98 (37) | 8 (3;14) |  |
| CLL / others | 34 (13) | 6 (3;18) |  |
| CML / MDS | 16 (6) | 7 (4;15) |  |
| Myeloma | 31 (12) | 8 (2;12) |  |
| BMT / HSCT recipient |  |  | 0.33 |
| Autologous | 39 (15) | 8 (3;17) |  |
| Allogenic | 40 (15) | 5 (1;13) |  |
| Disease status at admission |  |  | 0.54 |
| Newly diagnosed | 168 (68) | 8 (3;15) |  |
| Partial or complete remission | 78 (32) | 6 (2;16) |  |
| SOFA score | 219 | 0 (-0.13;0.12) | 0.97 |
| Mechanical Ventilation |  |  | 0.52 |
| Yes | 75 (29) | 8 (3;16) |  |
| No | 188 (71) | 8 (2;16) |  |
| Vasopressors |  |  | 0.34 |
| Yes | 95 (36) | 8 (3;17) |  |
| No | 168 (64) | 7 (2;15) |  |
| Renal replacement therapy |  |  | 0.18 |
| Yes | 36 (14) | 6 (2;11) |  |
| No | 224 (86) | 8 (3;18) |  |
| ICU LOS (days) | 263 | 0.14 (0.02;0.26) | 0.025 |

**Table S6. Association between different variables and Impact of Event Scale (IES) score 3 months after ICU discharge by univariate analysis.**
